# Methylmercury Exposure Induces Sexual Dysfunction in Male and Female *Drosophila Melanogaster*

**DOI:** 10.3390/ijerph14101108

**Published:** 2017-09-24

**Authors:** Ved Chauhan, Syian Srikumar, Sarah Aamer, Mirazkar D. Pandareesh, Abha Chauhan

**Affiliations:** New York State Institute for Basic Research in Developmental Disabilities, 1050 Forest Hill Road, Staten Island, NY 10314, USA; syians@bu.edu (S.S.); sarah.aamer@gmail.com (S.A.); pandareesh.d.mirazkar@opwdd.ny.gov (M.D.P.); abha.chauhan@opwdd.ny.gov (A.C.)

**Keywords:** copulation, *Drosophila melanogaster*, methylmercury, oxidative stress, sexual dysfunction

## Abstract

Mercury, an environmental health hazard, is a neurotoxic heavy metal. In this study, the effect of methylmercury (MeHg) exposure was analyzed on sexual behavior in *Drosophila melanogaster* (fruit fly), because neurons play a vital role in sexual functions. The virgin male and female flies were fed a diet mixed with different concentrations of MeHg (28.25, 56.5, 113, 226, and 339 µM) for four days, and the effect of MeHg on copulation of these flies was studied. While male and female control flies (no MeHg) and flies fed with lower concentrations of MeHg (28.25, 56.5 µM) copulated in a normal manner, male and female flies exposed to higher concentrations of MeHg (113, 226, and 339 µM) did not copulate. When male flies exposed to higher concentrations of MeHg were allowed to copulate with control female flies, only male flies fed with 113 µM MeHg were able to copulate. On the other hand, when female flies exposed to higher concentrations of MeHg were allowed to copulate with control male flies, none of the flies could copulate. After introduction of male and female flies in the copulation chamber, duration of wing flapping by male flies decreased in a MeHg-concentration-dependent manner from 101 ± 24 seconds (control) to 100.7 ± 18, 96 ±12, 59 ± 44, 31 ± 15, and 3.7 ± 2.7 seconds at 28.25, 56.5, 113, 226, and 339 µM MeHg, respectively. On the other hand, grooming in male and female flies increased in a MeHg-concentration-dependent manner. These findings suggest that MeHg exposure causes sexual dysfunction in male and female *Drosophila melanogaster*. Further studies showed that MeHg exposure increased oxidative stress and decreased triglyceride levels in a concentration–dependent manner in both male and female flies, suggesting that MeHg-induced oxidative stress and decreased triglyceride levels may partly contribute to sexual dysfunction in fruit flies.

## 1. Introduction

Among the heavy metals, mercury (Hg) is one of the most dangerous environmental pollutants [1]. The sources of Hg in the atmosphere include volcanoes, forest fires, and volatilization from the ocean. Since mercury occurs naturally in coal and other fossil fuels, it becomes airborne and releases into the atmosphere when people burn these fuels for energy. Metallic and inorganic Hg released into the atmosphere is brought down by rain, which is transformed to MeHg by anaerobic organisms in soil and water. MeHg then bio-accumulates in fish [2,3], which are later consumed by humans. In fish muscle, MeHg is predominantly present in the form of MeHg-L-cysteine (MeHg-L-Cys). Upon ingestion, gastrointestinal absorption of MeHg-L-Cys is faster as compared to that of MeHg, and it gets rapidly distributed to all tissues, including brain [4,5].

*Drosophila melanogaster* (fruit fly) is a widely used model for studies of brain disorders such as Parkinson’s disease, Alzheimer’s disease, Huntington’s disease, fragile X syndrome, and Angelman syndrome [6]. Among the 59 human neurological genes examined, 38 have orthologs in the *Drosophila* genome [7]. The *Drosophila* model exhibits complex behaviors relevant to human, including courtship [8], and grooming [9]. We have recently reported that bisphenol A, an environmental agent, affects the neurobehavior of fruit flies [10].

Sexual arousal is a complex phenomenon, which involves physiological, psychological, behavioral, and neural components [11,12]. Studies have suggested that environmental agents such as pesticides, industrial chemical pollutants, lead, and heavy metals have negative impact on human reproduction [13,14,15,16]. However, most of these studies are focused on the effect of environmental factors on male infertility and not much research has been done on their effect on sexual dysfunction in females.

The courtship behavior of the male *Drosophila melanogaster* has served as a good model for the study of the neural processes and computations that govern behavioral decisions. Under normal conditions, a male fruit fly will readily initiate courtship when placed in a chamber with a female fly, and follow a stereotyped sequence of courtship escalation [17]. First, a male fly recognizes a female fly and orients towards her. The male fly then taps the female fly on her abdomen with his forelegs, which have taste receptors. Courtship is then further escalated by the male fly’s wing extensions that vibrate to produce an auditory signal. The love song with wing flapping represents one of the most important information transfers from the male to the female fly [17] and is a large determinant of copulation success [18,19]. At this point, if the female fly is receptive to the advances of the male fly, he will lick her genitalia and immediately attempt to copulate. If unsuccessful, the male fly repeats the cycle, starting with a new song production [20]. The primary role of the central nervous system in the courtship song is the initiation and suppression of courtship activity [21]. The role of the female fly in courtship is to either accept or reject the male fly based upon how she perceives his courtship technique [8]. Over the course of being courted, the female fly decreases her locomotion, which allows the male fly to copulate with her [22]. The progressive decrease in the movement of the female fly during successful male courtship reflects a corresponding increase in the receptivity of the female fly [23].

Oxidative stress [24] and lipids [25] have been suggested to play important roles in the sexual dysfunction. Vigeh et al. [13,24] have suggested that lead–induced reproductive toxicity may be related to free radicals, i.e., reactive oxygen species (ROS). Environmental agents such as organophosphates have also been reported to induce production of ROS that lead to reproductive tissue damage [26]. In addition, polyunsaturated fatty acids (PUFAs) have also been suggested to play an important role in cellular signaling and sexual dysfunction in *Caenorhabditis elegans* and *Drosophila melanogaster* [25]. Drosophila have fat bodies, where triglycerides are the source of fatty acids. Therefore, excessive ROS may lead to sexual dysfunction by damaging fatty acids.

Since MeHg has neurotoxic effects and neurons are involved in sexual activities, we studied the effect of MeHg exposure on the behavioral parameters related to sexual function (copulation and wing flapping), oxidative stress, and triglyceride levels in male and female fruit flies.

## 2. Materials and Methods

*Drosophila melanogaster.* Wild-type Oregon-R *Drosophila melanogaster* stocks were maintained at 25 °C on a standard cornmeal diet (Jazz-mix *Drosophila* food, Fisher Scientific, Pittsburgh, PA, USA) under 12 h:12 h light and dark cycle.

*Chemicals.* Methylmercury chloride and all other chemicals for various assays were purchased from Sigma-Aldrich, St. Louis, MO, USA.

*Diet containing MeHg.* A stock of 100 mg MeHg/mL dimethylsulfoxide (DMSO) was prepared. Various concentrations of methylmercury chloride were mixed with 7 mL of food at final concentrations of 28.5, 56.5, 113, 226, and 339 µM MeHg. For controls (no MeHg), only DMSO was added to the diet.

### 2.1. Courtship Assays

Virgin male and female flies were collected within 6–8 h of emergence under light CO_2_ anesthesia and individually stored in food vials containing no MeHg (control) or different concentrations of MeHg (28.5, 56.5, 113, 226, 339 µM) for four days. For the observation of courtship behavior, one male fly and one female fly were aspirated without anesthesia into a courtship chamber (Aktogen, Cambridge, UK). They were kept isolated for 4–5 min before allowing them to interact with each other in various combinations of MeHg concentrations. The flies were video-recorded for 40 min. Two parameters of the courtship index were measured using time spent by male fly in wing flapping and the time taken by the male and female flies to come close for the first time to initiate copulation process within a 10 min observation period as described by Trott et al. [23].

### 2.2. Grooming

The grooming assay was performed as previously described [10]. For this assay, we used previously recorded videos for courtship assay and manually analyzed the grooming time episodes during the initial 10 min observation period.

### 2.3. Levels of Free Radicals, i.e., ROS

Total levels of ROS in the flies were measured using 2′,7′-dichlorofluorescein diacetate (DCFH-DA) as previously described [27,28]. Briefly, the frozen flies were sonicated with 1 mL of phosphate buffer (0.1 M; pH 7.4) and centrifuged at 10,000 *g* for 10 min at 4 °C. The supernatants were used to determine dichlorofluorescein (DCFH) oxidation as an index of ROS generation [29]. The protein concentration of the supernatants was determined by Bradford method [30]. In 96-well plates (black, flat-bottom), the supernatant (~50 µg protein) and 10 μL of DCFH (1.25 mM) were mixed and the volume of reaction mixture was adjusted to 200 µL with phosphate buffer, pH 7.4. The reaction mixture was incubated at 37 °C for 20 min and the fluorescence was measured at the excitation wavelength of 480 nm and emission wavelength of 530 nm using SpectraMax M5 multi-mode microplate reader (Molecular Devices, Sunnydale, CA, USA). The ROS levels are expressed as arbitrary fluorescence units/mg protein.

### 2.4. Lipid Peroxidation

Malondialdehyde (MDA) is an end product and marker of lipid peroxidation, and its levels were analyzed as described previously [31,32]. The flies frozen at −20 °C were homogenized in 1 mL of phosphate buffer (pH 7.0). To 0.25 mL of fly homogenate (~100 mg protein), 0.25 mL of trichloroacetic acid (10%) and 2 mL of thiobarbituric acid (TBA) mixture were added. The TBA mixture contained TBA (0.35%), sodium dodecyl sulfate (0.2%), FeCl_3_ (0.05 mM), and 0.5 mM butylated hydroxyl toluene in glycine-HCl buffer (100 mM, pH 3.6). The samples were incubated for 30 min in a boiling water bath and then allowed to cool. The samples were then centrifuged at 10,000 *g* for 10 min, and the absorbance of the supernatants was measured at 532 nm using SpectraMax M5 (Molecular Devices, Sunnydale, CA, USA) [32]. The MDA content in the samples was calculated by using the molecular coefficient for MDA at 1.56 × 10^5^.

### 2.5. Lipid Extraction

Male and female virgin flies were separated under CO_2_ anesthesia, and fed a diet containing different concentrations of MeHg for four days. Twenty male or twenty female flies frozen at −20 °C were homogenized in 300 µL of chloroform:methanol (2:1, V/V). After homogenization, 75 µL of water was added and vortexed, followed by centrifugation at 10,000 *g* for 10 min. The upper aqueous phase was discarded and the lower organic phase was used for the estimation of triglycerides and phospholipids.

### 2.6. Measurement of Triglyceride Levels 

Fifty microliters of organic phase and triglyceride standard were spotted on a silica-gel thin-layer chromatography plate. The plate was developed in a solvent system containing petroleum ether:ether:acetic acid, 90:10:1 (V/V). The triglyceride spot on the plate was scrapped and lipids were extracted twice with chloroform. The chloroform was evaporated and triglyceride levels were measured as described previously [33].

### 2.7. Measurement of Phospholipid Levels

Ten microliters of organic phase were dried and phospholipids were estimated as described previously [34].

### 2.8. Data Analysis

Data are presented as Mean ± Standard error of Mean (S.E.M) of four independent experiments. The statistical analysis of the data was performed by using GraphPad Prism 5 (GraphPad Software, Inc., La Jolla, CA, USA). The statistical significance between the experimental and control groups was examined by one-way ANOVA (Dunnette), and p values less than 0.05 were considered significant. To evaluate the correlation among MeHg-mediated changes in the levels of MDA, ROS, and triglycerides, linear regression analysis of the data was performed and Spearman correlation coefficient (r) was calculated.

## 3. Results

### 3.1. Effect of Different Concentrations of MeHg on the Initiation Time of Copulation

Individual male and female virgin flies were transferred to vials containing food with no MeHg (control), or with MeHg (28.25, 56.5, 113, 226, 339 µM). After four days, the male and female flies were introduced in various combinations into a courtship chamber (Table 1). The control flies copulated in 16.5 ± 3.6 min. Male and female flies exposed to lower concentrations of MeHg (28.25 and 56.5 µM) for four days copulated like normal flies with initiation time of 17 ± 2 and 13.7 ± 5 min, respectively. However, chronic treatment (12 days) with lower doses of MeHg (28.25, 56.5 µM) inhibited copulation (data not shown). On the other hand, acute exposure of male and female flies with higher concentrations of MeHg (113, 226, 339 µM) was also able to completely inhibit copulation. When MeHg-exposed male flies were allowed to copulate with female control flies, male flies exposed to MeHg (113 µM) could copulate with initiation time of 5.4 ± 1.4 min (Table 1).

### 3.2. Effect of Different Concentrations of MeHg in the Diet on the Duration of Wing Flapping in Male Flies

**Figure 1:**
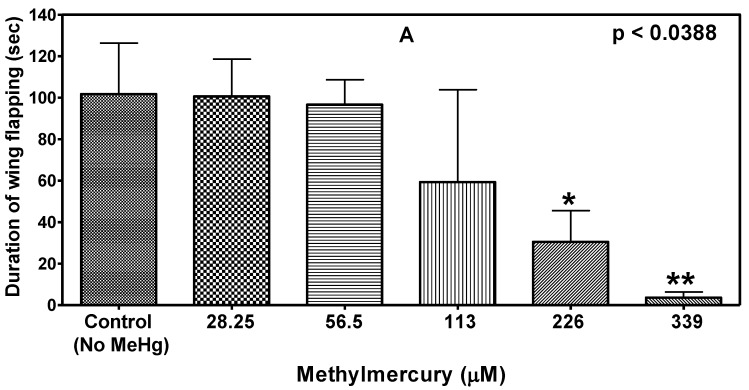
Effect of MeHg in the diet on duration of wing flapping by male flies. Duration of wing flapping during copulation was affected by MeHg exposure in a concentration-dependent manner (*p* < 0.0388, ANOVA). Data are presented as Mean ± S.E.M of four independent experiments. * *p* < 0.05, ** *p* < 0.01 as compared to controls.

The duration of wing flapping by a male flies during copulation was recorded as a function of MeHg concentration in the food. As shown in Figure 1, duration of wing flapping decreased in a MeHg concentration-dependent manner (*p* < 0.0388; ANOVA, Dunnette).

### 3.3. Effect of MeHg Exposure on the Grooming of Male and Female Flies in the Copulation Chamber

**Figure 2:**
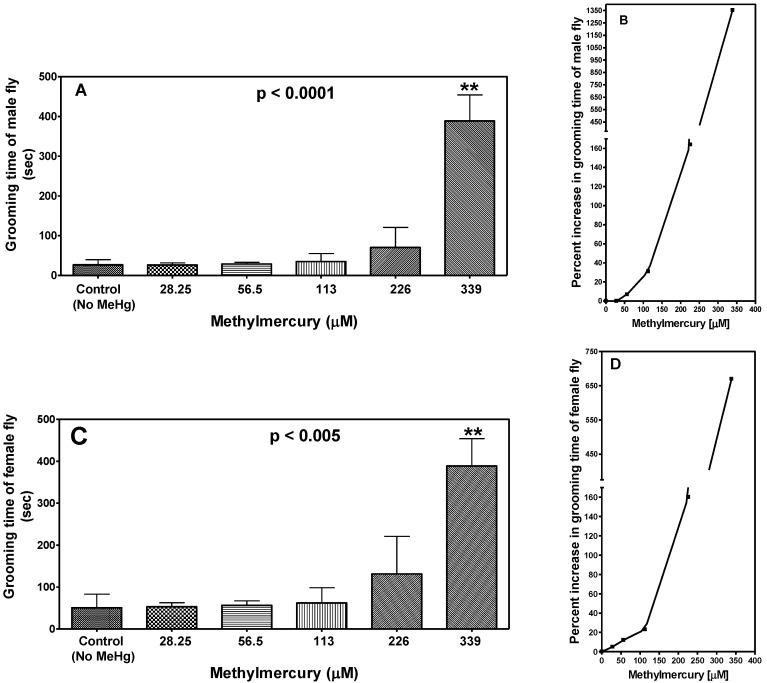
Effect of MeHg exposure on the grooming time in male and female flies. (**A**,**C**) represent MeHg-dependent effect on grooming time in male and female flies, and (**B**,**D**) show corresponding MeHg-induced percent increase in grooming time. Data are presented as Mean ± S.E.M of four independent experiments. ** *p* < 0.01 as compared to controls, ANOVA (Dunnette).

Total duration of grooming time (seconds) of male and female flies in the courtship chamber was manually analyzed in 10-min time periods using pre-recorded videos. As shown in Figure 2, duration of grooming time increased for both male (*p* < 0.0001) and female (*p* <0.005) flies in a MeHg concentration-dependent manner.

### 3.4. Effect of Different Concentrations of MeHg on Time Taken by Male and Female Flies to Come Close to Each Other in the Copulation Chamber

MeHg exposure in the flies did not have any significant effect on time taken to come closer in the copulation chamber at any of the concentrations studied, although flies exposed to higher concentrations of mercury, i.e., 226 and 339 µM, took more time than other flies (data not shown).

### 3.5. Methylmercury Decreases Triglyceride Levels in a Dose-Dependent Manner in Fruit Flies

**Figure 3:**
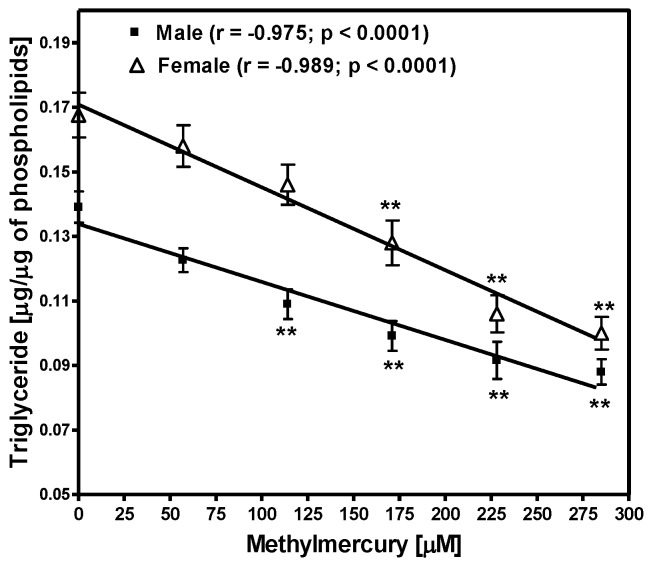
Effect of methylmercury exposure on the levels of triglycerides in male and female flies. Lipids were extracted from twenty male and twenty female flies fed different concentrations of MeHg for four days. After separation of triglycerides by thin layer chromatography, triglycerides levels were measured. Data are presented as Mean ± S.E.M of four independent experiments. ** *p* < 0.01 as compared to controls, ANOVA (Dunnette).

Because triglycerides are the main source of fatty acids in fruit flies, the levels of triglycerides were measured in flies fed with various concentrations of MeHg. There was a significant decrease in the levels of triglycerides in both male (*p* < 0.0001) and female (*p* < 0.0001) flies exposed to MeHg in a dose-dependent manner (Figure 3). Triglyceride levels were higher in females as compared to those in male flies. Linear regression showed a significant negative correlation between triglyceride levels in both male and female flies and MeHg concentrations in the diet (r = −0.975, male flies; and r = −0.989 in female flies).

### 3.6. Effect of MeHg on the Levels of Phospholipids in the Flies Fed with Different Concentrations of MeHg

The phospholipid’s phosphorus of the flies was analyzed in the organic phase of lipid extract. There was no effect of MeHg on the levels of phospholipids in the flies (data not shown).

### 3.7. Methylmercury Increases Oxidative Stress in a Dose-Dependent Manner in Both Male and Female Flies: Effect on Free Radicals (ROS) Generation and Lipid Peroxidation

**Figure 4:**
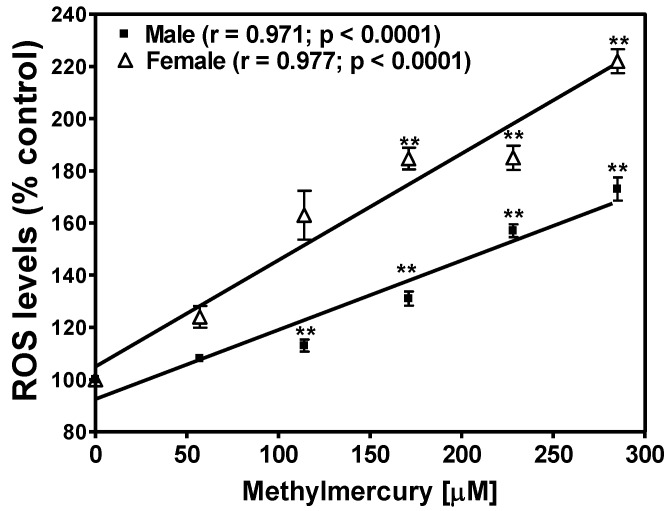
Effect of methylmercury in the diet on the levels of reactive oxygen species (ROS) in male and female flies. ROS values in control flies (no MeHg) were considered 100%. Data are presented as Mean ± S.E.M of four independent experiments. ** *p* < 0.01 as compared to controls, ANOVA (Dunnette). Linear regression analysis showed a significant correlation between methylmercury concentration in the diet and ROS levels in both male (r = 0.971) and female flies (r = 0.977).

**Figure 5:**
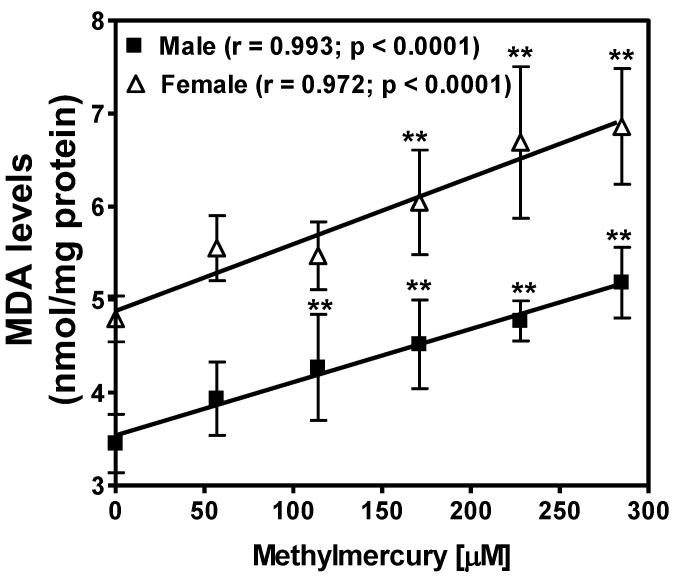
Effect of methylmercury on lipid peroxidation in male and female flies. Lipid peroxidation was assessed by measuring the levels of malondialdehyde (MDA), an end product of lipid peroxidation. Data are presented as Mean ± S.E.M of 4 independent experiments. ** *p* < 0.01, as compared to controls, ANOVA (Dunnette). Linear regression analysis showed a significant correlation between MeHg concentration in diet and MDA levels in both male (r = 0.993) and female flies (r = 0.972).

Diet supplemented with various concentrations of MeHg increased the levels of ROS in both male (*p* < 0.0001) and female flies (*p* < 0.0001) (Figure 4). MeHg exposure also increased lipid peroxidation assessed as MDA content in both male (*p* < 0.0001) and female flies (*p* < 0.0001) in a concentration-dependent manner (Figure 5). The MeHg-dependent increase in ROS and lipid peroxidation in females was higher as compared to that in male flies. This could be due to the presence of higher concentrations of triglycerides in females than male flies.

## 4. Discussion

Sexual dysfunction including low libido can be caused by lower levels of the estrogen hormone. The growth, metamorphosis, reproduction, and aging of *Drosophila* are controlled by fly’s steroid hormones known as ecdysteroids [35]. Active 20-hydroxyecdysones share structural similarities with mammalian estrogens. Fatigue, depression, anxiety, and certain medications such as antidepressants can also lead to low libido. Very little is known about how sexual dysfunction relates to low libido in male and females due to MeHg toxicity.

Our previous studies with bisphenol A have shown that *Drosophila* is a good model to assess behavioral abnormalities [10]. Our present study suggests that *Drosophila* is also an excellent model to study the male/female libido or other sexual dysfunctions. Our data suggest that copulation is affected in both male and female flies exposed to MeHg. When MeHg-fed male and female flies were allowed to copulate in the chamber, no copulation was detected in these flies exposed to higher concentrations of MeHg for four days (acute exposure) or to lower concentrations of MeHg for 12 days (chronic exposure), suggesting MeHg-induced sexual dysfunction in either male or female flies or in both male and female flies.

Our results suggest that MeHg decreases wing flapping by male flies. We measured two parameters for copulation index during a 10-min time period: (1) duration of wing flapping by male flies and (2) time taken by male and female flies to come close to each other to initiate copulation. Although flies exposed to higher concentrations of MeHg, i.e., 226 and 339 µM, took more time to come close to each other than did control and 133.7 µM MeHg-fed flies, there was no significant difference. Duration of wing flapping of male flies decreased in a MeHg concentration-dependent manner, suggesting that desire for copulation in male and female flies decreases as the concentration of MeHg increases. Since neurons and neuronal circuitry play important roles in the sexual functions [36,37], and MeHg induces neurotoxicity [38], it is possible that MeHg-induced sexual dysfunction is because of its neurotoxic effect.

Another reason for sexual dysfunction may be modulation of juvenile hormone and pheromones. In most female insects, the juvenile hormone regulates and coordinates reproductive maturation of ovaries [39,40], sex pheromone synthesis, and mating behavior [41]. In *Drosophila*, females are unreceptive to male courtship attempts just after eclosion, but by day two, most females become receptive and mate [42]. Further studies are required to understand whether MeHg affects juvenile hormone, pheromone, or receptiveness of females for mating.

It has been suggested that oxidative stress may cause reproductive tissue damage [13]. In order to understand the mechanism involved in sexual dysfunction induced by MeHg exposure, we studied the effects of MeHg on oxidative stress in male and female flies. Our results suggest that MeHg increases ROS and lipid peroxidation in a dose-dependent manner in both male and female flies, suggesting that oxidative stress may contribute in part to MeHg-mediated sexual dysfunction. ROS inhibits the production of sulfahydryl antioxidants, inhibits enzyme reactions, damages nucleic acids, and inhibits DNA repair [13]. ROS also damages polyunsaturated fatty acids. Our data also suggest that levels of triglycerides are decreased in a MeHg concentration-dependent manner in both male and female flies. Triglycerides are the main source of energy, which is generated by the fatty acids of triglycerides. Studies in *Drosophila* have implicated the roles of PUFAs and PUFA-derived signaling in reproduction and early developmental processes. The genome of flies has six Δ9 desaturase genes [43]. This proliferation of Δ9 desaturases is suggested to be an integral part for the production of gender and species-specific sex pheromones in *Drosophila* [43]. Sex pheromones in *Drosophila* are very long hydrocarbon chains that are secreted onto the cuticle and signal whether or not to engage in courtship behaviors. They are typically between 23 and 27 carbons and have 1 or 2 double bonds [44]. If PUFAs are an essential part of sex pheromones in *Drosophila*, it is possible that some of the effects of low libido in the flies exposed to MeHg may be the result of decreased triglyceride levels in these flies.

In fruit flies, hygienic grooming is induced by contact chemicals [45]. Our data also show that grooming time in flies fed with MeHg increases in a dose-dependent manner in both male and female flies, which could be related to hygienic grooming. It may be possible that sexual dysfunction in *Drosophila* may also be attributed to flies getting more involved in grooming, which may be diverting their attention from copulation. Sometimes chemicals may alter olfactory and gustatory pheromones of flies. Clowney et al. [46] reported that courtship ritual is triggered by activation of sexually dimorphic P1 interneurons. Gustatory and olfactory pheromones affect P1 neurons. MeHg may affect the olfactory and gustatory pheromones of flies, thus affecting the copulation process.

## 5. Conclusions

In conclusion, the exposure of environmental factors may play important roles in sexual dysfunction in both male and female flies. Courtship is escalated by male wing extensions that vibrate to produce an auditory signal. Our data suggest that MeHg-treated male flies have some desire for copulation as suggested by wing extensions. However, the wing extension is dramatically reduced in MeHg-treated male flies. It may be possible that reduction of wing extension is not sufficient to lure female flies for courtship. In the case of a female fly, her role in courtship is to either accept or reject the male based upon how she perceives his courtship technique [8]. A diet with MeHg also led to sexual dysfunction in female flies.

## Figures and Tables

**Table 1:** Time taken by flies to copulate in various groups.

Mating Groups	Initiation Time (min) of Copulation after Male and Female Flies Were Introduced in the Chamber
Control male (no MeHg) X Control female (no MeHg)	16.5 ± 3.6
28.25 µM MeHg-fed male X 28.25 µM MeHg-fed female	17.0 ± 2
56.5 µM MeHg-fed male X 56.5 µM MeHg-fed female	13.7 ± 5
113 µM MeHg-fed male X 113 µM MeHg-fed female	No copulation
226 µM MeHg-fed male X 226 µM MeHg-fed female	No copulation
339 µM MeHg-fed male X 339 µM MeHg-fed female	No copulation
Control male (no MeHg) X 113 µM MeHg-fed female	No copulation
Control male (no MeHg) X 226 µM MeHg-fed female	No copulation
Control male (no MeHg) X 339 µM MeHg-fed female	No copulation
Control female (no MeHg) X 113 µM MeHg-fed male	5.4 ± 1.4
Control female (no MHg) X 226 µM MeHg-fed male	No copulation
Control female (no MHg) X 339 µM MeHg-fed male	No copulation

Data are presented as Mean ± Standard error of Mean (S.E.M) of four independent experiments.
